# Health Service Utilization in Adolescents Following a First Arrest: The Role of Antisocial Behavior, Callous-Unemotional Traits, and Juvenile Justice System Processing

**DOI:** 10.1007/s10488-024-01341-x

**Published:** 2024-03-01

**Authors:** Julianne S. Speck, Paul J. Frick, Erin P. Vaughan, Toni M. Walker, Emily L. Robertson, James V. Ray, Tina D. Wall Myers, Laura C. Thornton, Laurence Steinberg, Elizabeth Cauffman

**Affiliations:** 1 Department of Psychology, Louisiana State University, 236 Audubon Hall, Baton Rouge, LA 70803 USA; 2 Harris County Juvenile Probation Department, Houston, USA; 3 Center for Child Behavior, Sarasota, USA; 4 University of Central Florida, Orlando, USA; 5 Eastern Louisiana Mental Health System, Jackson, USA; 6 Abt Associates, Austin, USA; 7 Temple University, Philadelphia, USA; 8 University of California, Irvine, Irvine, USA

**Keywords:** Health service utilization, Antisocial behavior, Juvenile justice system, Callous-unemotional traits

## Abstract

Previous research indicates that youth exhibiting antisocial behavior are at risk for utilizing a disproportionate amount of health services compared to youth without these problems. The present study investigates whether being processed by the juvenile justice system and showing callous-unemotional (CU) traits independently predict health service utilization (medical and mental health service use and out-of-home placement) over and above the severity of antisocial behavior across adolescence. A total of 766 participants who had been arrested for the first time in adolescence provided data at ten appointments over a period of seven years. Results showed that self-reported antisocial behavior at the time of arrest predicted increased use of most health service use types over the next seven years (i.e. medicine prescriptions, tests for sexually transmitted infections, mental health service appointments, and out-of-home placements). All except prescription medication use remained significant when controlling for justice system processing and CU traits. Further, justice system processing added significantly to the prediction of medical service appointments. Whereas CU traits were associated with mental health service appointments and out-of-home placements, these did not remain significant when controlling for severity of antisocial behavior. These findings are consistent with prior research documenting the health care costs of antisocial behavior.

The United States spends more per person on health care than any other country in the world (Anderson et al., 2019). This high cost has led to an increasing focus on factors that are related to “health service utilization”, which covers a range of services including dental care, medical care, mental health treatment, and out-of-home placement in hospitals, jails, and group homes. While much of the scrutiny has focused on inefficiencies in the health care system itself, there has also been interest in identifying characteristics that increase the quantity and cost of services that an individual will use over the life span, making certain groups of individuals a greater burden on the health system than others and making them an important target for preventive interventions.

Among these groups of interest are children and adolescents who display antisocial behavior and who are at risk for a host of problematic outcomes that could lead to higher health service utilization throughout the lifespan. Antisocial behaviors are defined as behavior that violates the basic rights of others (e.g. physical fights, cruelty, destruction of property, theft) or that violates major societal norms (e.g. truancy, running away from home; American Psychological Association, 2022). Problematic levels of antisocial behavior are present in between 3 and 7% of children and adolescents between the ages of 3 and 17 (Ghandour et al., 2019). Further, children demonstrating persistent or severe antisocial behaviors are at risk for a number of adverse mental health outcomes in adolescence and adulthood, including anxiety, depression, posttraumatic stress, and substance use disorders (Cyr et al., 2022; Kretschmer et al., 2014; Odgers et al., 2007). Studies have also shown that these children who show antisocial behaviors are at risk for a number of medical problems as well (e.g. Odgers et al., 2008; Paradis et al., 2016; Temcheff et al., 2023). Specifically, in a New Zealand birth cohort reassessed at age 32, antisocial behavior in childhood and adolescence predicted greater likelihood of contracting Type 2 herpes, chronic bronchitis symptoms, gum disease, and suffering serious injury (Odgers et al., 2008). Similarly, in a longitudinal study that followed 801 participants from age 7 to 42 in the Northeastern United States, children displaying elevated antisocial behavior were at greater risk for cardiovascular problems, lower back pain, cancer, and emergency department visits compared to those displaying no significant antisocial behavior (Paradis et al., 2016). Finally, children and adolescents exhibiting antisocial behavior are also at risk for criminal activity and involvement in the juvenile justice system (Erskine et al., 2016; Hofvander et al., 2017). In a meta-analysis of 13 outcome studies, children and adolescents demonstrating significant antisocial behavior were reported to have a 2.3–3.2 times increased likelihood of criminal activity in adulthood compared to non-antisocial youth (Bevilacqua et al., 2018).

As would be expected from the many adverse outcomes exhibited by children and adolescents who show persistent patterns of antisocial behavior, these youth use a disproportionate amount of health services (Costello et al., 2014). A nationally representative study of U.S. adolescents (*n* = 6483) demonstrated that youth with disorders defined by the presence of antisocial behavior [i.e. oppositional defiant disorder (ODD) and conduct disorder (CD)] were second only to youth with attention-deficit/hyperactivity disorder (ADHD) in terms of lifetime use of any mental health or medical service use, but that youth with ODD or CD diagnoses utilized the greatest proportion of “human services” (counseling, religious, or mental health crisis hotline help) and, alongside youth with substance use disorders, the greatest proportion of juvenile justice services (Merikangas et al., 2011). In the New Zealand cohort study cited earlier, children with serious antisocial behavior problems had 2–3 times the number of emergency department visits, 3–5 times the number of prescription medication fills, and 1.4–1.8 times the number of injury claims in adulthood compared to non-antisocial children (Rivenbark et al., 2018).

As noted previously, children and adolescents demonstrating serious antisocial behavior are at higher risk for involvement in the criminal justice system. Though the needs of adolescents in justice-involved settings are diverse and not limited to antisocial behavior (i.e. early-onset psychosis, trauma, internalizing problems, etc.), antisocial behavior that violates laws is what typically leads to juvenile justice system involvement. This link to justice system involvement is an important issue for understanding the service utilization of persons who show antisocial behavior, given that justice system involvement has itself been associated with high rates of mental and medical health needs (e.g. Barnert et al., 2016, 2017). A 2006 report from the National Center for Mental Health and Juvenile Justice in collaboration with the Council of Juvenile Correctional Administrators found that 70% of justice-involved youth met criteria for at least one mental health disorder (Skowyra & Cocozza, 2006). More recently, Livanou and colleagues (2019) conducted a meta-analysis to determine pooled prevalence rates for 22 diagnoses among justice-involved youth, founding that CD was the most common overall (66%; 95% CI: 56–76%) and that among males, the most common diagnoses were Antisocial Personality Disorder (81%; 95% CI: 69–91%), CD (68%; 95% CI: 56–79%) and Moderate Learning Disability (51%; 95% CI: 47–56%). These high rates of mental health needs for youth in the justice system continue into adulthood, as demonstrated by a 15-year follow-up study of 1,829 adults who had been incarcerated as juveniles that found that 64% of males still met criteria for a psychological disorder, including ADHD, CD, Major Depression, and Generalized Anxiety Disorder (Teplin et al., 2021). In addition to the high rate of mental health needs, there also appears to be a high rate of medical problems among youth served by the juvenile justice system. For example, youth in the juvenile justice system show a high rate of sexually transmitted diseases (Winkelman et al., 2017). Additionally, a study of over 7,000 adults from Germany on the health impact of incarceration reported that involvement with the juvenile justice system was associated with worse health outcomes, even after controlling for covariates such as schooling, intelligence, family income, and marital status (Schnittker & John, 2007).

While youth in the juvenile justice system have more mental and medical health needs than adolescents in the community, it is not clear if these needs are simply due to the previously established link between mental and medical health needs and antisocial behavior. Further, while there is evidence for increased medical and mental health needs of youth in the juvenile justice system and for greater service utilization, it is not clear that justice system involvement leads to more service utilization than would be obtained by youth displaying antisocial behavior who are not in the justice system. For example, an early study comparing the outcomes of adolescents recruited from either a detention facility, an inpatient psychiatric facility, or a community mental health clinic reported that incarcerated youth used more special education and residential services than those in the other groups but used less outpatient mental health and social services (Pumariega et al., 1999). Similarly, another study comparing juvenile justice-involved youth and youth involved with mental health treatment outside the justice system reported that justice-involved youth used fewer medical and mental health care services but more residential correctional services than youth involved in outpatient mental health treatment (Liebenberg & Ungar, 2014). These findings comparing justice and non-justice involved youth with mental health problems raise the possibility that justice-involved youth may not receive more services in general than other youth with significant mental health needs but may be more likely to be placed in out-of-home care, such as secure facilities, inpatient hospitals, or group homes, which are some of the costliest types of services (Espinosa et al., 2020). However, again, it is not clear if this use of expensive services can be accounted for by the severity of the adolescent’s behavior problems that brought them into contact with the justice system.

A final issue in advancing knowledge on the service utilization of youth with serious antisocial behavior is the fact that these youth vary greatly in the type, persistence, and severity of their behavior problems (Frick, 2012). An important subgroup of children displaying serious antisocial behavior that has emerged from recent research are those with elevated callous-unemotional (CU) traits. CU traits are defined by a lack of guilt, lack of empathy or concern for others, lack of concern about performance in important activities, and a shallow or deficient display of affect (Frick et al., 2014a). These traits are considered to represent the affective component of psychopathy in adults and the affective component of conscience in child samples (Frick et al., 2014b). CU traits are moderately correlated with antisocial behavior and are only elevated in a minority of antisocial youth (Frick et al., 2014a). However, they are critical for causal theories of antisocial behavior because they seem to differentiate subgroups of youth with serious antisocial behavior who have distinct genetic, emotional, and cognitive characteristics that could be indicative of different causal pathways to the development of antisocial behavior problems in those with and without elevated CU traits (De Brito et al., 2021).

Importantly, CU traits have also been associated with a number of problematic outcomes that could influence the service utilization of children exhibiting antisocial behavior who are elevated on these traits. Specifically, CU traits predict higher rates of self-reported delinquency (Kimonis et al., 2014), contacts with the police (Frick et al., 2005), violence (Baskin-Sommers et al., 2015), and arrests (Kahn et al., 2013; Kimonis et al., 2016). Thus, CU traits predict greater involvement with the juvenile justice system, which as noted above, can lead to greater utilization of some services, particularly expensive out-of-home placements. Further, CU traits have been associated with greater risk for other mental health outcomes, such as substance use, that can also increase their level of service utilization relative to other youth with antisocial behavior (Baskin-Sommers et al., 2015; Ray et al., 2016). Finally, CU traits are associated with risky sexual behavior even when controlling for their level of antisocial behavior (Thornton et al., 2019), which may also increase their health care utilization.

While there are reasons to believe that CU traits may increase the already high level of health care utilization associated with antisocial behavior, this has not been tested directly. Further, there is some research to suggest that the rate of utilization for some types of health services may not be higher for youth with elevated CU traits. Research on psychopathy broadly has indicated an association between psychopathic traits and negative health outcomes (e.g. Beaver et al., 2014; Meehan et al., 2019). As noted previously, CU traits form the affective component of psychopathy. In research on adults, this facet of psychopathy has been negatively associated with health problems (Hudek-Knežević et al., 2016; Međedović & Kujacic, 2020). Further, in a study of adolescent detainees, the affective facet of psychopathy was negatively related to anxious-depressive behavior (Sevecke et al., 2009). One possible explanation that has been proposed is that the affective dimension of psychopathy may be less associated with emotional distress in general (Međedović et al., 2018) and is associated with less physiological (Johnson et al., 2015) and behavioral responses to stress (Willemsen et al., 2012). Thus, while CU traits may be associated with some problematic outcomes, such as greater legal involvement and greater risk for antisocial outcomes, they may not be associated with greater risk for physical health outcomes. Therefore, CU traits may increase the risk for certain types of service utilization, such as mental health and out-of-home placement services, but they may not increase the risk for use of medical services, other than services for sexually transmitted diseases.

## The Current Study

In summary, while serious antisocial behavior problems have been linked to greater health service use, the independent roles of justice system involvement and CU traits in this link has not been studied. Thus, the present study seeks to disentangle the individual contributions of these three interrelated predictors of health service use in a sample of justice-involved male adolescents. Specifically, we tracked the usage of mental and physical health services through multiple follow-up assessments in the 7 years following the adolescent’s first arrest for an offense of mild to moderate severity. This design allowed us to have significant variability in both the number and severity of the youth’s antisocial behavior and in the percentage of youth who were formally processed after this first arrest (i.e. appeared before a judge) versus those who were diverted from the justice system after this first arrest (i.e. not formally adjudicated). This design allowed us to provide a strong test of the independent contribution of the severity of the adolescent’s antisocial behavior and the level of justice involvement on the youth’s service use over an extended period (i.e. 7 years). Further, we measured the youths’ level of CU traits at the time of arrest, using a well-validated self-report measure to test the contribution of these traits to the service use of the sample over adolescence and through the transition to young adulthood.

Using this design, we tested the following hypotheses. First, we hypothesized that the level of self-reported antisocial behavior (i.e. self-report of delinquency) would be positively associated with mental health service use, medical service use, and out-of-home placement over the follow-up period. Second, we hypothesized that formal processing after a youth’s first arrest would also increase the medical, mental health, and out-of-home placement service use compared to those who were diverted from the system. Third, we hypothesized that after controlling for antisocial behavior, processing type would no longer be associated with mental or medical health service use but would still predict out-of-home placement. Fourth, we hypothesized that elevated CU traits would predict mental health service use, tests for sexually transmitted diseases, and out-of-home placement independent of processing type and the level of self-reported antisocial behavior but would not be predictive of greater medical health service utilization.

## Method

### Participants

The present study utilized the sample collected for the Crossroads Study, a longitudinal project that investigated the effects of juvenile justice system involvement on a number of different outcomes (Cauffman et al., 2021). Recruitment of study participants involved cooperation between the probation department, district attorney’s office, and County or Parish Court at the participating sites (i.e. Orange County, CA; Jefferson Parish, LA; and Philadelphia, PA) to identify male youth between the ages of 13 and 17 who had been arrested for the first time for an offense of mild or moderate severity. Charges that led to inclusion in the Crossroads study were determined by examining court records over a 5-year period prior to study commencement. Using these historical records, charges were selected at each site for which youth with no prior offenses were formally processed in about 50% of the cases (charges that had a 0.35 to 0.65 probability of being formally processed). Thus, inclusionary charges were those where there was a great degree discretion on whether or not to formally process the youth. This study design was chosen to provide strong test of the effects of justice system involvement by only including youth with no prior offenses (and thus, with no previous justice system involvement) and youth with charges that had a substantial chance of being either formally or informally processed (and thus, having significant variability in this outcome).

Study enrollment resulted in a sample of 1,216 males (533 form Philadelphia, PA; 532 from Orange County, CA; and 151 from Jefferson Parish, LA) with a mean age of 15.29 (*SD* = 1.29). The sample recruited was racially and ethnically diverse, with Hispanic/Caucasian (45.9%), African American (36.9%), non-Hispanic/Caucasian (14.8%), and Other (2.5%) races and ethnicities represented. Participants’ intelligence was measured by the matrix reasoning and vocabulary sub-tests of the Wechsler Abbreviated Scale of Intelligence (WASI-II; Wechsler, 1999). These two subtests have been shown to produce scores highly related to full IQ test scores (Girard et al., 2015; Wechsler, 1999). This sample’s average estimated IQ was approximately 1 SD below the normative mean for the general population (*M* = 88.50, *SD* = 11.87). Using the child’s address and based on data from the 2013 American Community Survey administered by the United States Census Bureau, participant addresses were geocoded to census block groups, which represent the smallest geographic unit summarized by the census. Each participant’s address and corresponding block group was used to derive a value of neighborhood poverty, captured by the percentage of households falling below the poverty line within that block group.

### Procedures

Institutional Review Board approval for the Crossroads Study was obtained at the participating institutions prior to beginning data collection. Parental informed consent and youth assent were obtained at each time point for all participants before interviews were conducted until the youth turned 18 years old, at which point the parent or legal guardian was no longer asked to provide parental consent and the participant provided his consent. Research staff informed participants and their parents at each visit that participation was entirely voluntary, that it would not influence the youth’s relationship with the juvenile justice system or court, and that they may withdraw from the study at any time without penalty. The youth and parents were also informed that the research project had obtained a Privacy Certificate from the Department of Justice to protect their data from being subpoenaed for use in legal proceedings.

Youth completed the baseline assessment within six weeks of the disposition date for their initial arrest. They were then re-assessed every six months for 36 months (6 timepoints), yearly for the next two years (2 timepoints), and then at the 7-year follow-up. Interviews lasted on average approximately 2–3 h and were administered using a secure computer-based program on a laptop. Participants were able to select their preferred location to complete the interviews, often at the youth’s home, a local restaurant, public library, at the respective research team’s university, or in a secure facility if a participant was incarcerated at the time of a follow-up interview. Phone interviews were completed in the event of natural disaster (e.g. hurricanes) or the COVID-19 pandemic or if the participant had moved too far away to conduct an in-person appointment. The retention rate ranged from 95.5% at the 6-month follow-up to 76.2% at the 7-year follow-up. The average retention rate across the 8 follow-up timepoints was 91.2%.

### Measures—Baseline Predictors

#### Antisocial Behavior

The Self-Report of Offending Scale (SRO) was administered at the baseline assessment to collect information on the adolescent’s self-reported lifetime engagement in a range of antisocial behaviors (Huizinga et al., 1991). For each of the 24 items, participants responded if they had ever engaged in each crime and these responses were summed for a total score of the number of different offenses the participant had committed in their lifetime up until the first study visit. Self-report of antisocial behavior typically captures more instances of this behavior compared to official report of arrests, though they are often highly correlated (e.g. Pollock et al., 2015). Use of a variety score instead of a frequency score prevents the resulting composite from being highly influenced by frequent but less severe offenses (Sweeten, 2012). Also, the variety score from the SRO has been correlated with official records of criminal offending in adolescent samples (Thornberry & Krohn, 2000). The Cronbach’s alpha of the SRO in the current sample was $\alpha$ = 0.82.

#### Callous-Unemotional Traits

CU traits were assessed at baseline using the self-report version of the Inventory of Callous-Unemotional traits (ICU), a 24-item instrument that utilizes a four-point Likert scale (i.e. 0 – Not at all true to 3 – Definitely true) to have the adolescent indicate how accurately each statement describes him. While the ICU items have been found to factor in several sub-domains, the items consistently load onto an overarching factor that is captured well by unit weighing of items, the subscales are largely the result of method variance (i.e. positive vs. negatively worded items), the subscales show variance that is primarily due to the overarching factor, and the subscales do not show consistent and theoretically meaningfully differential associations with important external criteria (Kemp et al., 2022; Ray & Frick, 2020; Ray et al., 2016). Additionally, concurrent and predictive validity of the total ICU score has been demonstrated by positive associations with antisocial behavior and negative associations with prosocial behavior across a range of adolescent samples (Cardinale & Marsh, 2020). Thus, only the total ICU score collected at baseline was used in current analyses. Cronbach’s alpha was $\alpha$ = 0.76 for the ICU in the present study.

#### Juvenile Justice System Processing

Youth were divided into two groups based on how they were processed by the justice system after their first arrest. One group consisted of formally processed youth, whose cases were petitioned and went through the formal court system, resulting in court-ordered probation or adjudication. The other group consisted of informally processed youth, who were diverted from court and were handled only by a probation department or other designated agency (e.g. Families in Need of Supervision or a mental health agency). Justice system processing type was coded as “0” for informally processed youth and “1” for formally processed youth.

### Measures—Outcome Variables

#### Health Service Utilization

Health service utilization was assessed at every timepoint through a self-report survey. At each follow-up assessment, the participant was asked about the number of appointments for medical services (i.e. doctor or nurse checkups, dentist visits, emergency room visits, and ambulance rides to a hospital) and mental health services (i.e. seen a therapist or psychiatrist, attended an outpatient substance use clinic, received treatment from a therapist because of drug use, and seen a therapist for a substance use disorder) they had attended since the last assessment. Additionally, participants were asked at each appointment if they had taken a test for a sexually transmitted infection (STI) and how many prescription medications they had taken. Four variables were used in analyses: the number of medical service appointments, the number of prescription medications the participant reported taking, the number of appointments at which they reported having had an STI test, and the number of mental health service appointments over the course of the study. For each of these variables, a total score was used that summed the applicable data reported for each participant across all follow-up points, creating four continuous variables of health service use: three pertaining to medical service use and one pertaining to mental health service use. Adolescents have been shown to be valid reporters of health service use, with studies finding significant correlations between adolescent-report of service use and mental health diagnoses (Benjet et al., 2016), academic and social functioning (Ranta et al., 2009), school absenteeism (Askeland et al., 2015), and other indicators of need for services, such as past criminal behavior and substance use problems (Mulvey et al., 2007).

#### Out-of-Home Placement

At each follow-up assessment, participants provided information about their places of residence using a month-by-month calendar. This calendar captured the number of days the youth spent in various living situations since the previous assessment point. Categories of out-of-home placement included secure institutions (e.g. state prisons, local jails, secure state juvenile facilities, secure private provider facilities, secure drug or alcohol treatment program, or a juvenile hall), group homes or other supervised living communities, residential treatment centers, medical hospitals, psychiatric hospitals, and shelters. Life history calendars have been used in past research with adolescents (e.g. Fisher, 2013; Whitbeck et al., 1999) and young adults (e.g. Caspi et al., 1996; Freedman et al., 1988) to assist with recall for a variety of events, including living situation. These studies demonstrate that life history calendars pose benefits such as improved recall and accuracy of recall compared to standard surveys. The results of these studies suggest that adolescents are valid reporters of a variety of experiences including living arrangements (Whitbeck et al., 1999), household moves (Luke et al., 2011, 2012), and time spent in secure institutions (Schubert et al., 2016). A total score of the number of months spent in out-of-home placement over all follow-ups were calculated for each participant.

### Analytic Plan

#### Missing Data

Due to the different length of follow-up periods and the use of outcomes that were highly dependent on the time period assessed (i.e. number of doctor visits since last interview, amount of time in out-of-home placements since last interview), only participants who complete all 9 follow-up assessments were included in analyses. This led to a final sample of *N* = 766, which was 63% of the original sample of 1,216. Chi-square tests indicated differences in processing type ($\chi$ ^2^(1) = 4.40, *p* < 0.05, $\phi$ = − 0.06), Black race ($\chi$ ^2^(1) = 6.58, *p* < 0.05, $\phi$ = − 0.07), and Hispanic ethnicity ($\chi$ ^2^(1) = 3.25, *p* > 0.05, $\phi$ = 0.05) such that the subsample included in the present analyses showed lower rates of Black, Hispanic, and formally processed participants than the group who did not complete all follow-up assessments. A one-way ANOVA was conducted to compare those who completed all assessments with those who did not on continuous demographic variables (age, IQ, neighborhood poverty) and baseline predictors (SRO, ICU). The results of these analyses indicated that there were significant differences between the groups on age at baseline appointment (F(1, 1214) = 11.84, *p* < 0.001, $\eta$ ^2^ = 0.01), IQ (F(1, 1213) = 14.52, *p* < 0.001, $\eta$ ^2^ = 0.01), and neighborhood poverty (F(1, 1203) = 10.36, *p* < 0.01, $\eta$ ^2^ = 0.01), such that participants in the subsample used in the present analyses were older at the baseline appointment, had higher IQ scores, and lived in neighborhoods with slightly fewer households falling below the poverty line. It is important to note that the effect sizes for these differences in the follow-up and original sample, while statistically significant, were generally quite small.

***Analyses.*** The main study hypotheses were tested using series of linear multiple regression analyses, controlling for age at baseline, race/ethnicity, IQ, and neighborhood poverty. The hypotheses were tested through a series of regression models, with each outcome tested separately. That is, to test the first hypothesis, self-reported antisocial behavior was included as a predictor of service use variables controlling for all covariates. By entering the covariates into the model in the first step, it was then possible to estimate the predictive utility of study variables of interest independent of the variability in the outcomes associated with the covariates. To test the second hypothesis, these analyses were repeated with processing type (formally processed vs. diverted) used as the predictor. To test the third hypothesis, both self-reported antisocial behavior and processing type were included together with covariates in the regression model. Finally, to test the fourth hypothesis, CU traits were included with the covariates and both other predictors to determine its independent contribution to the prediction of the service use outcomes.

## Results

### Preliminary Analyses

Descriptive information and zero-order correlations for major study variables are presented in Table 1. As shown in this table, antisocial behavior was significantly positively associated with most measures of health service utilization with the exception of medical service use. Processing type was significantly positively associated with medical service use and CU traits were significantly positively associated with mental health service use and out-of-home placements.

**Table 1:** Descriptive statistics and correlations among measures

	N	M	SD	1	2	3	4	5
Demographic Variables								
1. Age	766	15.38	1.27	–				
2. Black	766	34%	–	− .09**	–			
3. Hispanic	766	48%	–	.00**	− .69**	–		
4. IQ	765	89.40	11.62	.05**	− .17**	− .07**	–	
5. NP	764	24.49	18.06	− .12**	.30**	− .09**	− .23**	–
Baseline Predictors								
6. SRO	766	3.35	2.97	.22**	− .13**	.07**	.10**	− .12**
7. Processing Type	766	43%	–	− .03**	− .02**	.09**	− .07**	− .00**
8. ICU Total Score	766	25.96	8.11	− .01**	− .08**	.13**	− .08**	.04**
Health Service Outcomes								
9. Medical Service Appointments	760	30.38	21.46	− .08**	.12**	− .11**	− .01**	.05**
10. Prescription Medications Appointments	760	3.11	3.96	.01**	− .09**	− .05**	.20**	− .13**
11. STI Test Years	753	4.18	2.96	.13**	.34**	− .23**	.00**	.14**
12. Mental Health Appointments	760	10.79	22.11	− .12**	.06**	− .13**	.05**	.04**
13. OOHP	766	2.91	6.38	− .10**	.14**	− .10**	− .08**	.05**

	6	7	8	9	10	11	12
Baseline Predictors							
6. SRO	–						
7. Processing Type	.05**	-					
8. ICU Total Score	.33**	.03*	-				
Health Service Outcomes							
9. Medical Service Appointments	.07**	.08*	.04**	–			
10. Prescription medications appointments	.11**	.02*	.05**	.46**	–		
11. STI Test Years	.12**	.06*	.06**	.31**	.10**	–	
12. Mental Health Appointments	.10**	.00*	.09**	.30**	.31**	.16**	–
13. OOHP	.11**	.07	.10**	.14**	.10**	.20**	.40**

Zero-order correlations between variables. Race, ethnicity, and processing type variables were dichotomously coded as “0” for individuals who did not identify as Black or Hispanic and those who were informally processed and “1” for those who identified as Black or Hispanic and were formally processed through the justice system. Percentages reported for these variables are the percent of individuals identified as Black, Hispanic, and formally processed. *IQ* intelligence quotient, *NP* neighborhood poverty, with values representing the percentage of households falling below the poverty line, *SRO* self-report of offending, *ICU* Inventory of Callous-Unemotional Traits, *STI* sexually transmitted infections, *OOHP* out-of-home placement

^*^p < .05

^**^p < .01

### Main Analyses

The results of the simultaneous multiple regression analyses in which the demographic variables and self-reported antisocial behavior predicted the various health service utilization outcomes are reported in Table 2. As shown in Table 2 and consistent with our hypotheses, self-reported antisocial behavior significantly predicted all service utilization outcomes after controlling for the covariates. The standardized Beta, which can be used as an effect size estimate, ranged from $\beta$ = 0.09 (*p* < *0.0*5) for predicting use of prescription medications to $\beta$ = 0.16 (*p* < *0.0*1) for predicting out-of-home placements.

**Table 2:** Simultaneous multiple regression analyses with self-reported delinquency predicting service utilization outcomes

	Medical service appointments	Prescription medications	STI tests	Mental health service appointments	OOHP
Age	− .10**	− .03**	.14**	− .15**	− .13**
Black	.06**	− .13**	.40**	− .08**	− .11**
Hispanic	− .08**	− .14**	.04**	− .19**	− .03**
IQ	− .01**	.15**	.07**	.03**	− .07**
NP	.02**	− .06**	.08**	.06**	.01**
SRO	.11**	.09**	.14**	.14**	.16**
Model R^2^	.03**	.06**	.17**	.06**	.06**

Coefficients reported are standardized Beta from ordinary least square regression equations. *STI* sexually transmitted infection, *OOHP* out-of-home placement, *IQ* intelligence quotient, *NP* neighborhood poverty, *SRO* self-report of offending

^*^p < .05; **p < .01

The results of analyses for the second hypothesis, in which justice system processing is tested as a predictor of the health service use outcomes over and above the demographic variables, are reported in Table 3 and show partial support for the hypothesis. That is, processing type predicted two of the three variables of medical service use: medical service appointments ($\beta$ = 0.09, *p* < 0.05) and STI test use ($\beta$ = 0.08, *p* < 0.05). However, contrary to hypotheses, processing type did not predict use of prescription medication, mental health service use, or time spent in out-of-home placements.

**Table 3:** Simultaneous multiple regression analyses with processing type predicting service utilization outcomes

	Medical service appointments	Prescription medications	STI tests	Mental health service appointments	OOHP
Age	− .07**	− .01**	.17**	− .12**	− .09**
Black	.05**	− .14**	.38**	− .10**	.09**
Hispanic	− .09**	− .14**	.04**	− .19**	− .04**
IQ	.00**	.16**	.08**	.04**	− .06**
NP	.02**	− .07**	.07**	.05**	.00**
Processing Type	.09**	.05**	.08**	.01**	.06**
Model R^2^	.03**	.06**	.16**	.04**	.03**

Coefficients reported are standardized Beta from ordinary least squares regression equations. *STI* sexually transmitted infection, *OOHP* out-of-home placement, *IQ* intelligence quotient, *NP* neighborhood poverty

^*^p < .05

^**^p < .01

These results testing the third hypothesis, in which both antisocial behavior and justice system processing type are included in the model predicting service use outcomes, are presented in Table 4. Contrary to our hypotheses, STI test use and medical service appointments continued to be significantly predicted by justice system processing when controlling for self-reported antisocial behavior ($\beta$ = 0.07, *p* < 0.05; $\beta$ = 0.08, *p* < 0.05; respectively). Further, antisocial behavior remained significantly associated with all service use outcomes after controlling for covariates and justice system processing.

**Table 4:** Linear multiple regression analyses with processing type and self-reported delinquency predicting service utilization outcomes

	Medical service appointments	Prescription medications	STI tests	Mental health service appointments	OOHP
Age	− .10**	− .03**	.14**	− .15**	− .13**
Black	.06**	− .13**	.39**	− .08**	.10**
Hispanic	− .09**	− .14**	.03**	− .19**	− .04**
IQ	− .00**	.15**	.07**	.03**	− .07**
NP	.02**	− .06**	.08**	.06**	.01**
Processing Type	.08**	.04**	.07**	.01**	.05**
SRO	.10**	.09**	.14**	.14**	.16**
Model R^2^	.04**	.07**	.17**	.06**	.06**

Coefficients reported are standardized Beta from ordinary least squares regression analyses. Processing type was included in the model in the first step and self-reported delinquency was added in the second step. *STI* sexually transmitted infection, *OOHP* out-of-home placement, *IQ* intelligence quotient, *NP* neighborhood poverty, *SRO* self-report of offending

^*^p < .05

^**^p < .01

Lastly, the regression analyses testing the fourth hypothesis, in which CU traits are included in the model, are summarized in Table 5. Contrary to our hypotheses, CU traits did not add to the prediction of any health service utilization variable when controlling for self-reported delinquency and justice system processing.

**Table 5:** Linear multiple regression analyses with processing type, self-reported delinquency, and callous-unemotional traits predicting service utilization outcomes

	Medical service appointments	Prescription medications	STI tests	Mental health service appointments	OOHP
Age	− .09**	− .03**	.15**	− .15**	− .12**
Black	.06**	− .13**	.39**	− .08**	.10**
Hispanic	− .09**	− .15**	.03**	− .20**	− .05**
IQ	− .00**	.16**	.08**	.04**	− .06**
NP	.02**	− .06**	.07**	.05**	.01**
Processing Type	.08**	.04**	.07**	− 01**	.05**
SRO	.10**	.07**	.12**	.11**	.14**
ICU Total	.02**	.06**	.05**	.07**	.06**
Model R^2^	.04**	.07**	.18**	.06**	.06**

Coefficients reported are standardized Beta from ordinary least squares regression analyses. *STI* sexually transmitted infection, *OOHP* out-of-home placement, *IQ* intelligence quotient, *NP* neighborhood poverty, *SRO* self-report of offending, *ICU* Inventory of Callous-Unemotional Traits

^*^p < .05

^**^p < .01

## Discussion

Our results support and add nuance to a large amount of past research showing the high costs associated with antisocial behavior in adolescents (e.g. Rivenbark et al., 2018). That is, self-reported delinquency predicted all types of health service utilization over a 7-year period that covered most of adolescence and the transition to adulthood. These results support past research showing that youth demonstrating antisocial behavior are at risk for a number of poor mental health, physical health, and legal outcomes (e.g. Odgers et al., 2008; Temcheff et al., 2023) and, as a result, tend to utilize more health services than other youth (e.g. Eijgermans et al., 2021; Merikangas et al., 2011).

Our results advance this work by testing whether this association with antisocial behavior accounted for the greater service use demonstrated by persons in the juvenile justice system or whether justice system involvement adds to the prediction of service use, even when controlling for a youth’s level of antisocial behavior. That is, past research has shown that involvement in the juvenile justice system is associated with greater mental health and medical service use and in particular, out-of-home placement, when compared to youth in the community (Barrett et al., 2006; Brown et al., 2020; Pumariega et al., 1999). However, it was not clear from this past work whether this link could be solely accounted for by the higher level of antisocial behavior displayed by those involved in the juvenile justice system. Our results suggest that the youth’s level of antisocial behavior appears to be the stronger predictor for most service use outcomes, especially the expensive use of out-of-home placements. That is, being formally processed after arrest in our sample only predicted greater medical service use and use of STI tests after controlling for covariates and self-reported delinquency. These findings support the need to control for the level of antisocial behavior when considering the impact of justice system involvement on certain types of health care use, which is critically important given that the vast majority of youth in the justice system show serious antisocial behavior problems (Beaudry et al., 2021; Teplin et al., 2021).

Another important advance of the current study is that it also tested whether CU traits uniquely contributed to the prediction of health service use, beyond the level of antisocial behavior shown by the adolescent and the degree of justice system involvement. As predicted, CU traits were not associated with greater medical service uses. Also, consistent with hypotheses, CU traits were associated with greater use of mental health services and more out-of-home placements. However, neither of these associations remained significant when controlling for youth’s level of antisocial behavior. Though there is a growing body of research showing that CU traits are an indicator of additional impairment for youth with serious antisocial behavior that is not solely accounted for by their level of antisocial behavior (e.g. McMahon et al., 2010; Thornton et al., 2013), our findings suggest that CU traits do not necessarily put youth at greater risk for use of health services. Service use seems more related to their level of antisocial behavior.

The current study had a number of strengths. Most importantly, the sample was large and designed to result in high numbers of youth who were formally processed and youth who were diverted from the justice system, with crimes generally of similar severity. This design allowed for a strong test of study hypotheses, given that it resulted in a sample with great variability on all three predictors of interest (i.e. antisocial behavior, justice system involvement, and CU traits). Additionally, we employed a large number of follow-up assessments (i.e. 9 time points) over an extended period (i.e. 7 years), which provided a very strong assessment of health care utilization across adolescence and into the transition to adulthood.

However, the study results should also be interpreted in light of several limitations. First, though the sample was large and racially and ethnically diverse, participants were all male and all of whom had been arrested, thus our findings do not necessarily generalize to samples of girls and community samples. Second, due to different time windows across the follow-up period and the way health care utilization was measured (e.g. number of appointments since last interview, time spent in placements since last interview), only participants who completed all assessment were used in the analyses. This led to a sample used in analyses that differed from the full sample, although these differences had small effect sizes. Third, all measures except for justice system involvement were collected via self-report, which may have led to inflated correlations due to shared method variance. Fourth, the total variance accounted for in these models was modest, ranging from *R*^*2*^ = 0.05, *p* < 0.01 for medical appointment service use to *R*^*2*^ = 0.18, p < 0.01 for STI test use. These effect sizes indicate that there are many other relevant factors that were not included in our study that contribute to health service utilization in justice-involved adolescents, such as additional mental health and behavioral problems that increase health service use (e.g. anxiety, depression, substance use, post-traumatic stress disorder), access to healthcare (e.g. access to reliable transportation, health insurance coverage and income), availability of services in an individual’s community, and attitudes towards health care systems and providers.

Within the context of these limitations, our results add to the call for more funding to enhance the prevention and treatment of antisocial behavior. As noted by Rivenbark et al.’s (2018) 30-year follow-up of a birth cohort in New Zealand, persons displaying childhood antisocial behavior problems represented only 9% of birth cohort but accounted for 50% criminal convictions, 15% of hospital bed nights, 16% of emergency department visits, 21% of prescription fills, 13% of injury claims, and 25% of welfare benefit months used for the sample in adulthood. Thus, this relatively small group of individuals accounted for a substantial proportion of health service usage. Further, there are a number of interventions that have been proven effective in preventing and reducing serious antisocial behavior in children and adolescents (McMahon et al., 2021), with estimates suggesting that the benefit of a targeted intervention for problems with antisocial behavior leads to a return of investment of about $7 to $21 for every $1 used for the intervention (Cohen & Piquero, 2015). Despite this great cost of antisocial behavior and the tremendous potential benefit of prevention, these behavior problems are not given priority in mental health treatment funding for children and adolescents (Burt et al., 2018).

Finally, our results support the need to consider the child’s level of antisocial behavior when investigating the effects of other variables on an adolescent’s health care utilization, such as CU traits and juvenile justice involvement. For example, our results did not find that the justice system had some of the anticipated iatrogenic effects on health care utilization (e.g. greater out-of-home placements), which appeared to be largely due to the adolescent’s level of antisocial behavior. We did find that adolescents who were formally processed after their first arrest used more medical services. However, it is unclear whether or not the increased medical service use is a positive indicator of justice system involvement (i.e. youth were getting needed services) or a negative indicator (i.e. youth were being exposed to violence that necessitates medical treatment; e.g. Beck & Rantala, 2016). Even if there is an increase in obtaining needed medical services for youth in the justice system, it is not clear that this benefit would outweigh potential harmful effects of such involvement (Cauffman et al., 2021). As a result, it would be important to find other ways to link youth with significant antisocial behavior to needed health care services that don’t place them at risk for some of the harmful effects of juvenile justice system involvement (e.g. exposure to antisocial peers; increased risk for exposure to violence; Cauffman et al., 2021).
